# Defending the right to health in Gaza: a call to action by health workers

**DOI:** 10.1186/s13031-024-00613-5

**Published:** 2024-09-20

**Authors:** Fatima Mohammed, Umniha Siddig Ahmed Elgailani, Sondos Yassir Ibrahim Ali, Razan Faisal Abdullah Mohamed, Elaine Tan Su Yin, Martha L. Bravo-Vasquez

**Affiliations:** 1 Faculty of Medicine, Khartoum University, Khartoum, Sudan; 2 Faculty of medicine and health sciences, Omdurman Islamic University, Omdurman, Sudan; 3 Faculty of Medicine, Al Neelain University, Khartoum, Sudan; 4 Faculty of Medicine, Elrazi University, Khartoum, Sudan; 5 School of Medicine, Zhejiang University, Hangzhou, China; 6 Universidad Nacional Mayor de San Marcos, Lima, Peru

**Keywords:** Conflict, Attacks, Inequities, Malnutrition, Health system

## Abstract

The ongoing conflict in Gaza has led to severe destruction of the health system and eventually its collapse. Moreover, multiple attacks on health workers were reported which led to obstacles in service delivery. The conflict has led to further humanitarian crises including shortage of food, water sanitation, and hygiene, and outbreaks of infectious diseases. About 2 million of Gaza’s population are internally displaced with the majority in Rafah. Rafah’s population has increased by 500% in less than four months. This has led to acute food severity in Gaza for the whole of the population. Moreover, the cut of UNRWA aid is expected to further expand the humanitarian crisis as over 2 million of the population depends on the aid.

## Attacks on the health system in Gaza

The access to health services in Gaza has been compromised, even before the events of the 7th of October 2023. There have been different restrictions on the access to healthcare services of Palestinians in Gaza by the Israeli authorities [1]. From January to July 2023, 189 attacks were reported on healthcare in The Occupied Palestinian Territory, 10 of them in the Gaza Strip, and 152 health workers were directly affected [2].

Since the 7th of October, the war in Gaza has worsened the health system crisis, which has led to a growing humanitarian catastrophe that threatens the collapse of Gaza and has taken an unbelievable and intolerable toll. There is evidence of persistent assaults on medical workers, facilities, and transportation as there have been at least a total of 443 healthcare attacks reported, and 447 in the West Bank [3]. These attacks have led to the death of 480 health workers and arrest of injuries, and arrest of 160. [4, 5]. The attacks involved various forms of violence, including physical assault, disruption of healthcare delivery, criminalization, arrest and detention of patients and healthcare workers, and collapsing Gaza’s health system, especially service delivery, medical personnel, and facilities [6]. Only 12 out of 36 hospitals in Gaza are partially functioning because of power outages, fuel shortages for generators, or attacks like airstrikes, or a significant shortage of health workers [7]. For instance, the closure of the Gaza Strip’s only oncology hospital over a fuel shortage has placed around 2,000 cancer patients in danger [8]. The closure of maternity hospitals like Al Hilo Hospital resulted in 180 women giving birth without essential medical supplies every day [9].

Furthermore, the high bed occupancy rates of up to 150% of their total capacity, making infection prevention and control nearly impossible due to broken water and sanitation systems, running out of cleaning materials, doctors are forced to perform surgeries without lights and anesthesia [8]. The complicated logistics of transportation, damaged roads, and the inability to deliver fuel and vital medical supplies to major hospitals contribute to the difficulty of mobilizing essential life-saving materials [8]. Subsequently, the intense overcrowding and disrupted health, water, and sanitation systems in Gaza exacerbated infectious disease outbreaks over short time frames, as the number of deaths and injuries continued to rise. More than 71,000 cases, 8944 cases of scabies and lice, 1005 cases of chickenpox, and 54,866 cases of upper respiratory infections have been recorded since the start of the war [10].

Moreover, the risk of accelerated disease spread is further increased by interrupted routine vaccination programs and a shortage of medications for treating infectious diseases over extended periods. This, in turn, exacerbates the disease burden over an extended period. Another contributing factor is the disease surveillance system’s insufficient coverage of early disease detection and response capabilities [10].

## The current humanitarian situation in Rafah

Internal displacement accounts for more than 75% of Gaza’s population of 2 million people. In Rafah, a small strip of land with a population five times larger than before the war, over a million people live in cramped conditions [11].

The medical system is in dire straits. As sanitation deteriorates, hospitals are overcrowded, and medical staff are working under extreme stress and hardship. Unimaginable obstacles stand in the way of the estimated 5,500 expectant mothers who will give birth in the upcoming month in getting access to quality medical care; the majority of them no longer have the facilities required to give birth safely [11].

Rafah’s population has increased by 500% in less than four months, making it one of the most populated areas of Gaza even before October 7. Dangerous illnesses like smallpox, hepatitis A, diarrhea, and lice are becoming more widespread. The United Nations (UN) reports that one in ten children under the age of five are acutely malnourished, indicating that the prevalence of malnutrition is rising [12].

Given the ongoing prevalence of hunger and disease among the displaced population, any further decline in Rafah’s humanitarian situation would be disastrous. There was no running water, showers, or personal hygiene supplies available to the 27,400 civilians staying in nine shelters in Rafah. Numerous displaced individuals passed the night on the streets while the shelters were filled to 150% of their capacity [13].

Based on conditions in Gaza between November 24, 2023, and December 7, 2023, the most recent Integrated Food Security Phase Classification (IPC) report determined that acute food insecurity in Gaza is at “crisis or worse” for the entire population. About 378,000 people, or 15% of the population, are in Phase 5 (catastrophic levels), and 939,000 people, or 40% of the population, are in Phase 4 (emergency levels). According to IPC projections, by February of this year, food insecurity is expected to reach previously unheard-of levels [14].

### UNRWA funding cut

The United Nations Agency for Palestinian Refugees (UNRWA), which is thought to be a lifeline for two million people living in the besieged enclave, experienced funding cuts on Friday, January 26, following allegations by Israel that several of its employees were involved in the October 7 Hamas attack [15]. With 58 authorized refugee camps, UNRWA offers support, advocacy, and protection to almost 5 million officially registered Palestinian refugees in the Middle East [16].

Over two million civilians in Gaza, more than half of whom are children, depend on UNRWA relief; the suspension of financing by donor states will affect this life-saving support [17]. According to the UNRWA statement, the people in Gaza face malnutrition, an impending famine, and an outbreak of diseases as a result of Israel’s ongoing indiscriminate bombardment and deliberate refusal of assistance [17].

Furthermore, the WHO stated that over 14,000 people in the Gaza Strip require medical evacuation, and the great majority of them have war-related injuries [18]. According to WHO more than 1,200 patients, which equals to 50 patients per day, have been unable to leave Gaza to receive the needed medical treatment abroad [18].

### Successful interventions and possible solutions to healthcare delivery in conflict zones

In conflict zones worldwide, ensuring access to adequate healthcare remains a formidable challenge, exacerbated by the complexities of war and displacement. Nonetheless, innovative approaches have emerged to address the urgent healthcare needs of affected populations.

This discourse delves into successful interventions and potential solutions for healthcare delivery in conflict zones, with a particular focus on Gaza. Collaborative efforts between humanitarian organizations and local authorities, including the delegation of tasks to indigenous groups, have proven effective in regions such as Afghanistan, Somalia, Yemen, Mali, and Syria. For instance, in Syria, international humanitarian agencies partnered with local non-governmental organizations (NGOs), overseeing their operations through remote communication and visits by Syrian healthcare workers to nearby cities like Gaziantep or Amman. It not only facilitated enhanced data collection but also embraced innovative methods such as eHealth platforms and telecommunication [19].

Moreover, ongoing eHealth initiatives in conflict zones have identified three primary domains: clinical management, healthcare education, and information management. Notable projects like the Aga Khan-Tech4Life eHealth interventions in Afghanistan equipped community health workers with mobile applications containing health information recording systems, enabling efficient monitoring of health data across various epidemiological parameters. This transition to handheld devices with versatile capabilities, rather than relying solely on extensive infrastructure, aligns with the technological landscape of conflict zones where mobile technology is prevalent despite lacking infrastructure [20].

Furthermore, mobile clinics have emerged as vital components of healthcare delivery, especially for Internally Displaced Persons in areas like Northwest Syria. Through comprehensive case studies, it is evident that the deployment of mobile clinics follows a systematic process involving pre-deployment planning, design, execution, and feedback. Despite persisting challenges in regions like Northwest Syria, such as discrepancies in supply and demand and varying acceptance of mobile clinics, stakeholders continue to navigate these hurdles in the pursuit of improved healthcare access [21].

These successful interventions and potential solutions offer valuable insights for addressing healthcare delivery challenges in Gaza’s current situation. By fostering collaborative approaches, harnessing innovative eHealth solutions, and deploying mobile clinics strategically, stakeholders can navigate the intricate landscape of healthcare provision and ultimately enhance the well-being of affected populations amidst the ongoing conflict and displacement.

### Envisioning the reconstruction of Gaza’s healthcare system

The study of healthcare systems recovery in conflict- and crisis-affected states has been done by analyzing the cases of countries from Sub-Saharan Africa and the Middle-Eastern region like Afghanistan and Cambodia. These cases allow us to identify potential difficulties to rebuild Gaza’s healthcare systems and policy recommendations to overcome them. First, the government must create a long-term national plan that includes effective and impartial monitoring and evaluation mechanisms to ensure the constant improvement of their healthcare system even after the external help leaves and during the time NGOs and international missions are present, it could sign regional contracts to implement a Basic Package of Health Service with funding from the major donors (World Bank, WHO, and USAID) [22]. Second, social programs should be implemented in order to ensure universal access to health, some examples are health equity funds and community-based health insurance or covering a wider range of access costs than just facility fees, and ensuring non-discriminatory care for users [23]. Third, the healthcare system will need human resources to work, then distribution of personnel might be needed, but also the creation of incentives to attract and retain health professionals which could be done by offering high remunerations, providing space and flexibility and hiring foreign workers [23].

Furthermore, a multinational alliance of over 150 organizations came together in November 2023 to address the problems with healthcare brought on by the most recent Gaza war at the 1st International Conference to Rebuild the Health Sector in Gaza in Amman, Jordan [24].

A plan for rebuilding Gaza’s healthcare system was put forth during the Amman meeting, with a focus on vulnerable populations like children, the elderly, expectant mothers, individuals with chronic illnesses, patients in need of immediate surgery, medical professionals, and students pursuing health-related fields. The three temporal frames of the vision—immediate humanitarian needs, recovery trajectory, and thorough health system reconstruction—were based on the limited data and assumptions that were available [24].

The following challenges were discussed including restoring clean water sources, clearing roads, and ensuring health professionals’ safety. Moreover, the proposed recovery plans were divided into mid-term and long-term components. For instance, the mid-term recovery phase will focus on restoring services, while the long-term reconstruction phase will involve building and equipping secondary and tertiary hospitals and universities. In addition, external healthcare staff will be deployed to relieve Palestinian healthcare professionals and support emergency trauma surgery, and the human resources support will focus on training the next generation of healthcare workers [24].

## A call to action

As healthcare professionals, we strongly disagree with the ongoing attacks on health facilities and staff, which compromise the accessibility to health services by civilians, especially women and children who are affected the most. We call on the non-profit organizations dedicated to medical assistance, such as Médecins Sans Frontiéres (MSF), The International Committee of the Red Cross (ICRC), Save the Children, to forge a coalition to safeguard healthcare workers in Gaza. These entities are pivotal in rectifying the damage to the healthcare infrastructure in Gaza and fortifying the healthcare personnel’s resilience. Furthermore, we implore the United Nations member states to strategically advocate for the adherence to human rights principles, and prioritize health above political benefits. Such advocacy fosters an environment where dignity and well-being are protected during warfare. In this critical time, we need to stand in steadfast solidarity collectively in championing the cause of humanity. Only through concerted international action, we can aspire to alleviate the suffering endured by the innocent civilians in Gaza and work towards a future where the universal right to accessible healthcare is unequivocally upheld. Lastly, we call for an immediate and permanent ceasefire in Gaza that allows to work towards the reconstruction of the country and its health system.

## Data Availability

No datasets were generated or analysed during the current study.
